# Multiple Pathways for Protein Transport to Peroxisomes

**DOI:** 10.1016/j.jmb.2015.02.005

**Published:** 2015-03-27

**Authors:** P.K. Kim, E.H. Hettema

**Affiliations:** 1 Program in Cell Biology, Hospital for Sick Children, Toronto, ON, Canada M5G 1X8; 2 Department of Biochemistry, University of Toronto, Toronto, ON, Canada M5S 1A8; 3 Department of Molecular Biology and Biotechnology, University of Sheffield, Firth Court, Western Bank, Sheffield, South Yorkshire S10 2TN, United Kingdom

**Keywords:** PMP, peroxisomal membrane protein, PTS, peroxisomal targeting signal, TA, tail-anchored, peroxisome, peroxin, protein import

## Abstract

Peroxisomes are unique among the organelles of the endomembrane system. Unlike other organelles that derive most if not all of their proteins from the ER (*e*ndoplasmic *r*eticulum), peroxisomes contain dedicated machineries for import of matrix proteins and insertion of membrane proteins. However, peroxisomes are also able to import a subset of their membrane proteins from the ER. One aspect of peroxisome biology that has remained ill defined is the role the various import pathways play in peroxisome maintenance. In this review, we discuss the available data on matrix and membrane protein import into peroxisomes.

## Highlights

• Peroxisomal membrane and matrix proteins require distinct factors for their transport.
• Matrix proteins fold in the cytosol prior to their import.
• Loaded targeting receptors form part of the matrix protein translocation pore.
• Many membrane proteins are directly inserted into the peroxisomal membrane.
• Some peroxisomal membrane proteins are transported via the ER to peroxisomes.

## Graphical Abstract

**Figure:**
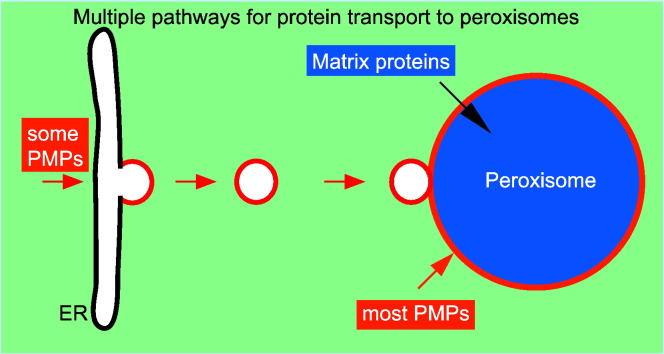

## Introduction

Peroxisomes are organelles found in almost all eukaryotic cells. They are bounded by a single membrane and are usually spherical. Peroxisomes are involved in a variety of metabolic pathways that vary between species, cell types and environmental conditions. All peroxisomes contain enzymes that catalyze oxidation reactions, including a fatty acid oxidation system, as well as enzymes that protect cells from oxidative damage, such as catalase. It is not surprising that peroxisomes are essential for human metabolism and development, and this is illustrated by severe inherited diseases caused by impairments in one or more peroxisomal functions [1], [2].

Peroxisomes are unique among single-membrane organelles in that peroxisomes are capable of importing both their matrix and membrane proteins directly from the cytosol. Peroxisomal matrix proteins are nuclear encoded and are synthesized on free polyribosomes [3]. As is the case for other targeting and translocation systems, peroxisomal matrix proteins contain specific targeting signals that are recognized by receptors that deliver them to the membrane translocation site. Translocation is dependent on consumption of energy in the form of ATP. Several aspects of peroxisomal protein transport distinguish it from other translocation systems. For example, peroxisomal proteins can fold, acquire cofactors and assemble into oligomers in the cytosol prior to import; another difference relates to the targeting receptors, which bind cargo in the cytosol but imbed into or cross the membrane as part of their targeting cycle.

Peroxisomal membrane proteins (PMPs) are also nuclear encoded. However, the mechanism of their import into peroxisomes is less understood. What is known is that PMPs and matrix proteins use different machineries for import. Most peroxisome biogenesis mutants contain membrane structures that are devoid of content, reflecting the large number of proteins required for import of matrix proteins (Table 1). The proteins required for peroxisome biogenesis are called peroxins (PEX) [4]. Only two PEX mutants in yeast (and three in mammals) are devoid of peroxisomal membrane structures.

Below, we review the targeting and import of matrix proteins separately from that of membrane proteins. We will discuss some of the discrepancies in the literature and the outstanding questions in the field. For detailed discussions on peroxisome multiplication, we refer to recent reviews [5], [6], [7], [8], [9].

## Matrix Protein Import

**Fig. 1:**
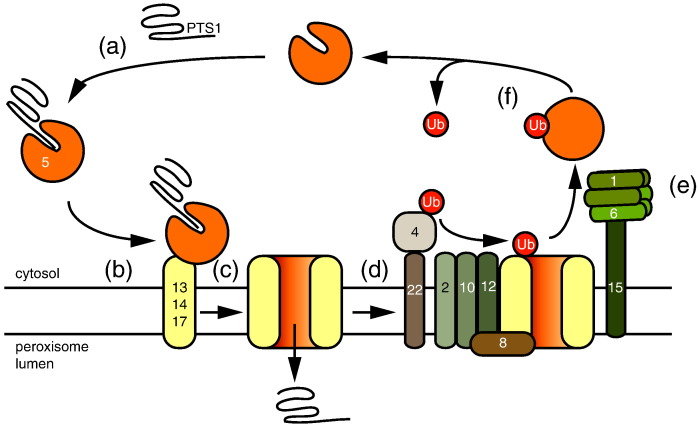
Model for import of peroxisomal matrix proteins containing a PTS1 in *S*. *cerevisiae*. (a) A newly synthesized PTS1-containing peroxisomal matrix protein is recognized in the cytosol by the receptor Pex5. (b) Cargo-loaded receptor docks onto the docking complex consisting of Pex13, Pex14 and Pex17. (c) Receptor inserts in the membrane in complex with Pex14 and cargo is released into the lumen. (d) The ubiquitin ligase complex (Pex2, Pex10 and Pex12) in conjunction with the ubiquitin conjugation complex (Pex4 and Pex22) modifies Pex5 on a cysteine residue. (e) Ubiquitinated Pex5 is extracted from the membrane by the extraction complex (Pex1, Pex6 and Pex15) and (f) subsequently deubiquitinated for another round of targeting.

Import of peroxisomal enzymes can be divided in multiple steps: (1) recognition in the cytosol by receptors, (2) docking of receptor–cargo complex at the peroxisomal membrane, (3) cargo translocation and (4) recycling of receptors (see Fig. 1).

As mentioned above, matrix protein import into peroxisomes differs from import into other organelles in that peroxisomes can import folded proteins and even protein complexes.

## Folding Prior to Import

Studies in the 1980s revealed that mRNAs encoding peroxisomal enzymes are associated with free polyribosomes [3]. Further evidence for posttranslational import is based on various experiments: (1) radioactive pulse-chase analyses show that newly synthesized β-oxidation enzymes are released in the cytosol before associating with peroxisomes [3], (2) *in vitro* synthesized peroxisomal matrix proteins are imported into purified peroxisomes (e.g., see Refs. [10], [11], [12]) and (3) import of purified peroxisomal proteins occurs in semi-permeabilized fibroblasts and after microinjection into the cytoplasm [13], [14], [15]. Indeed, the presence of a carboxy-terminal targeting signal in most peroxisomal matrix proteins is compatible only with a posttranslational targeting mechanism.

Whereas proteins targeted posttranslationally to mitochondria, chloroplasts and ER (*e*ndoplasmic *r*eticulum) are kept in a partially unfolded conformation by chaperones and targeting factors, peroxisomal matrix proteins can fold and assemble into their active, cofactor-containing conformation in the cytosol before targeting (for a review, see Ref. [16]). For example, catalase is a haem-containing tetrameric protein. In fibroblasts from patients with a peroxisome biogenesis defect, catalase is mislocalized to the cytosol. Fusion of peroxisome-deficient cells belonging to different complementation groups (i.e., having defects in different genes) results in import of the existing cytosolic pool of catalase [17]. Several studies have shown import of proteins that were folded prior to import [14], [15], [18]. Subunits of a homodimer or homooligomers artificially lacking a peroxisomal targeting signal (PTS) are co-imported with subunits containing a PTS [19], [20], [21], [22], [23]. Large structures (including gold particles or chemically cross-linked albumin) are imported into peroxisomes when coated with peptides containing a PTS after microinjection into the cytosol of fibroblasts [24].

However, folding is not a prerequisite for import [25], and not all peroxisomal proteins are multimers. Although tetrameric catalase can be imported *in vivo* [17], [23], an *in vitro* study reported that catalase monomers preferentially bind the import receptor that inhibits oligomerization. This would suggest that catalase is imported mainly as a monomer if the concentration of the receptor is high enough to bind newly synthesized catalase [26]. Likewise, pumpkin isocitrate lyase monomers are imported preferentially over tetramers *in vitro* [27]. Other oligomeric proteins that assemble into their quaternary structures inside peroxisomes include *Hansenula polymorpha* alcohol oxidase (an octamer of 600 kDa) [28], [29] and porcine heart peroxisomal carbonyl reductase (a tetramer). The latter protein cannot be imported as a tetramer as the PTSs are no longer accessible for recognition by the PTS receptor [30].

Therefore, although peroxisomes are capable of importing fully assembled oligomeric proteins, this may not reflect the way these proteins are imported under physiological conditions.

## Recognition by Targeting Receptors

The carboxy-terminal PTS1 was identified first in firefly luciferase and has since been found in most matrix proteins. The C-terminal tripeptide SKL was found to be both necessary and sufficient to target a protein to peroxisomes [31]. Variations of this signal have been described, resulting in the consensus -(SAC)-(KRH)-(LM) that is evolutionarily conserved [32], [33], [34], [35]. Some PTS1s do not conform to the consensus, and amino acid residues immediately preceding the tripeptide appear to contribute to targeting in those cases [36], [37], [38]. The PTS1 is recognized by the receptor Pex5, which is a modular protein containing a C-terminal tetratricopeptide repeat domain: it is the tetratricopeptide repeat domain that interacts with the PTS1 [39], [40].

A second evolutionarily conserved PTS is found close to the amino-terminus of a subset of peroxisomal matrix proteins. This PTS2 was first described as a nonapeptide present in the amino-terminal cleavable presequence of rat peroxisomal thiolase [41], [42], [43]. Most PTS2s fit the following consensus -R-(LIVQ)-X-X-(LIVQH)-(LSGA)-X-(HQ)-(LA)- [43]. The PTS2 is recognized by the receptor Pex7. The PTS2-dependent targeting route is absent in *Caenorhabditis elegans*, *Drosophila melanogaster* and diatoms [44], [45], [46].

Whereas Pex5 is sufficient for targeting PTS1-containing proteins to the peroxisomal membrane, many fungi require the coreceptor Pex20 for PTS2 targeting via Pex7 [47], [48], [49] or the Pex20-like paralogues Pex18 and Pex21 in *Saccharomyces cerevisiae* [50]. These coreceptors are absent from mammals and plants, where PTS2 import requires both Pex7 and Pex5. Human cells express at least two isoforms of Pex5: Pex5S and Pex5L. The latter has an additional exon that is required for binding of Pex7 to Pex5. This binding site is conserved in the Pex20-like proteins and plant Pex5 and is required for PTS2 import [47], [51], [52], [53]. Besides the Pex7 binding sites, the carboxy-terminal domain of the Pex20-like proteins shows additional sequence similarities to the N-terminus of Pex5. Therefore, Pex5L is considered a Pex7 coreceptor.

Not all peroxisomal matrix proteins are targeted to peroxisomes under all conditions. For instance, the activity of the PTS2 of *S*. *cerevisiae* GPD1 depends on phosphorylation [54].

## Non-Classical Import Mechanisms

Interestingly, not all matrix proteins contain a PTS1 or a PTS2 [20]. Cu/Zn superoxide dismutase 1 is found mainly in mitochondria and cytosol but is also localized to peroxisomes. This non-PTS1/PTS2-containing protein is piggy back imported into peroxisomes through its direct interaction with the PTS1-containing copper chaperone for superoxide dismutase [22].

Others, however, are not imported via the classical PTS1, PTS2 or even by a piggy-back pathway. Instead, proteins such as acyl-CoA oxidase (Pox1) in *S*. *cerevisiae* are recognized via the N-terminal domain of Pex5 rather than via its PTS1 binding domain [55] (for review, see Ref. [56]).

## The Recycling Receptor Concept

Early analysis of human Pex5 revealed it to be localized mainly to the cytosol, with a small fraction associated with peroxisomes. Localization to peroxisomal membranes is increased in the skin fibroblasts from peroxisome biogenesis disorder patients with a mutation in Pex2, Pex10 or Pex12 and in wild-type cells upon ATP depletion and incubation at low temperature. On restoring translocation conditions, Pex5 returned to the cytosol, after which it could be trapped again on peroxisomes by depleting ATP and incubating at low temperature. These experiments show clearly that Pex5 is a cycling receptor and that this cycle is modulated by factors required for peroxisomal protein import [57].

Different models have been proposed for Pex5 shuttling between cytosol and peroxisomal membrane, including a model whereby it dips into the membrane and releases its cargo at the luminal side of the membrane (shuttle model) [58] and another model whereby Pex5 enters the peroxisomal lumen before being exported back to the cytosol (extended shuttle model) [59]. Pex7 also behaves similar to a cycling receptor and was proposed to follow the extended shuttle model [60]. It is clear from these studies that Pex5 and Pex7 are exposed to the lumen of the peroxisome, but whether they are released into the lumen is not clear.

## Docking of Cargo–Receptor Complexes

The docking complex consists of Pex13 and Pex14 (and Pex17 in yeasts) and is required for both PTS1 and PTS2 import [61]. The docking complex proteins interact with the PTS receptors and each other (for detailed reviews, see Refs. [62], [63], [64]). The exact roles of Pex13 and Pex14 in docking are unclear. Both proteins have been shown to interact with cargo-loaded PTS1 and PTS2 receptors [65], [66], [67]. In mammals, cargo-loaded PTS1 receptor associates first with Pex14 before being translocated to a high-molecular-weight complex containing Pex13 [68]. The cargo-loaded PTS2 receptor complex also docks initially onto Pex14 via its coreceptor Pex5L [65], [69]. Subsequently, the Pex7-cargo-loaded complex binds to Pex13 independently of Pex5L [65], [67]. In *S*. *cerevisiae*, the PTS2 receptor complex appears to dock first onto Pex13 [66].

## Cargo Translocation and Release

After docking of the cargo-loaded receptors, the cargo must be translocated across the membrane and released inside the peroxisome. These processes are poorly understood. *In vitro* import studies using Pex5 are starting to give new insights into the translocation mechanism, which needs to allow for translocation of oligomeric proteins without disturbance of the internal milieu of the peroxisome. Several observations are compatible with a model whereby translocation occurs through a dynamic transient pore that disassembles after translocation. This pore is proposed to be composed of the targeting receptors plus components of the docking complex [70]. Indeed, Pex5 behaves as a soluble protein in the cytosol, as well as a carbonate-extraction-resistant and partially protease protected protein when in its cargo-loaded form at the peroxisomal membrane: as a receptor–cargo complex, Pex5 becomes an integral part of the translocation pore and behaves as an integral membrane protein. The C-terminal domain containing the PTS1 binding site is then exposed to the lumen of the peroxisome [71], [72], [73]. Pex5 has been reported to form homooligomers and to insert spontaneously into membranes [74], [75].

Pex5 together with Pex14 are the only peroxins required for import of Pex8 into peroxisomes, and they therefore constitute the minimal translocon [76]. *In vitro*, the *S*. *cerevisiae* Pex5–Pex14 complex can form an ion-conducting channel with a variable pore size of up to 9 nm [77], with the size of the pore depending on the size of the cargo. *Leishmania* Pex14 has also been shown to have pore-forming activity [78]. The actual architecture of the pore is unknown, and many questions remain, such as the driving force for translocation, whether PTS2 proteins are transported via the same or a separate pore and whether the Pex7 coreceptors will form part of the pore.

The mechanism of release of cargo from receptors has remained elusive. Recent *in vitro* studies suggest that Pex14 in mammals and Pex8 in *Pichia pastoris* may be involved [26], [79]. In plants, Pex14 has been suggested to be required for release of PTS2 cargo [80]. Furthermore, binding/release of cargo to Pex5 has been suggested to be redox dependent [79]. The development of an *in vitro* import system that follows the import of a specific cargo rather than that of the targeting receptors [81] is an important step that will likely resolve some of the mysteries.

## Export of Receptors

After release of their cargo, the receptors are exported back to the cytosol in an ATP-dependent process [68], [72], [82]. The export of receptors has been reviewed recently in Ref. [83] and will be discussed only briefly below.

PTS1 import requires monoubiquitination of a conserved cysteine in Pex5 [84], [85], [86]. Monoubiquitination occurs after insertion of Pex5 into the membrane and cargo release [87] and is essential for recycling of Pex5 and therefore for PTS1 import [85], [86], [88]. Monoubiquitination is catalyzed by the dedicated ubiquitin-conjugating enzyme Pex4 that is localized to the cytosolic side of the peroxisomal membrane [86], [88], [89]. In mammals, Pex4 is absent, and Pex5 monoubiquitination depends on a family of related ubiquitin-conjugating proteins (Ubc5a–Ubc5c) [90]. Pex5 monoubiquitination also depends on an integral membrane protein complex containing the RING ubiquitin ligases Pex2, Pex10 and Pex12 [91], [92], [93], [94].

*In vitro* export assays revealed that monoubiquitinated Pex5 is exported by the AAA-type ATPases Pex1 and Pex6 [68], [72], [95]. The hexameric AAA + complex consists of three subunits each of Pex1 and Pex6 [96]. The complex associates at the cytosolic side of the peroxisomal membrane via the tail-anchored (TA) protein Pex15 in yeast, Pex26 in mammals and APEM9 in *Arabidopsis thaliana* [97], [98], [99].

The mechanism by which monoubiquitinated Pex5 is recognized by Pex1/Pex6 is unknown. However, a novel binding partner for Pex6 comprises the ubiquitin-binding adaptor protein AWP1 [100], and it is tempting to speculate that this protein may be involved in recognition of monoubiquitinated Pex5. This suggests that Pex5 export is mediated by being pulled/extracted from the cytosolic side of the peroxisomal membrane. Therefore, at later stages of the import process, Pex5 is exposed to the cytosol where it is monoubiquitinated prior to its export. After release, Pex5 is deubiquitinated by the ubiquitin hydrolase Ubp15 in yeast and USP9X in mammals [101], [102], and it is ready for another round of cargo targeting and import. Import of proteins across membranes always requires energy. The only steps requiring energy are the monoubiquitination of Pex5 and its extraction from the membrane by Pex1 and Pex6 AAA + proteins. This led to the export-driven import model proposing that Pex1 and Pex6 function as motor proteins that couple ATP-dependent removal of Pex5 with cargo translocation into the organelle [103].

Analogous to the recycling of Pex5, the PTS2 coreceptor proteins have been shown to recycle by a mechanism requiring ubiquitination [49], [93]: they are monoubiquitinated on a cysteine residue near the amino-terminus, and this is dependent on Pex4 and the RING complex [93], [104], [105]. In mammals, Pex7 association with peroxisomes is dependent on Pex5L and cargo. The protection of Pex7 from protease digestion suggests its insertion deep in the membrane or its release into the lumen. ATP is required late in the cycle, after Pex7 and cargo become protease protected. Export of Pex7 requires Pex5L, but differences in the kinetics of export of Pex5L compared to Pex7 suggest that they may leave separately [106].

## Membrane Protein Targeting

The machinery required for PMP import is distinct from that required for matrix protein import. In almost all patients with peroxisome biogenesis disorders, matrix proteins are mislocalized to the cytosol, while PMPs are present in membrane structures called peroxisomal membrane ghosts [17], [107], [108]. The exceptions to this are two mutants in yeasts (*pex3* and *pex19*) and three mutants in mammals (*pex3*, *pex16* and *pex19*) that lack peroxisomal membrane ghosts: PMPs no longer colocalize with each other and are either rapidly degraded or localized to the cytosol or to membranes including mitochondria or putative pre-peroxisomal structures [109], [110], [111], [112], [113], [114], [115], [116], [117], [118], [119]. For this reason, Pex3, Pex16 and Pex19 are thought to be involved in the formation of peroxisomal membranes and/or insertion of PMPs into peroxisomal membranes.

Biochemical and microscopy studies of *pex3*, *pex16* and *pex19* mutants suggest that at least two routes exist by which PMPs can reach peroxisomes: one direct route and one via the ER. Below, we discuss the various routes and machineries involved in PMP insertion and provide examples to illustrate that a PMP may not be confined to a single route.

## Direct Targeting Pathway

Most PMPs are synthesized on free polyribosomes and imported posttranslationally from the cytosol [120], [121], [122], [123], [124], [125], [126]. Many of these PMPs possess a membrane PTS (mPTS) that consists of a cluster of positively charged residues or a mixture of positively charged and hydrophobic residues flanked by one or two transmembrane segments that are recognized and bound by Pex19 [127], [128], [129], [130], [131], [132], [133], [134], [135], [136], [137]. However, there is another group of PMPs that does not bind Pex19 (Jones *et al*., 2004). Thus, PMP membrane targeting signals have been classified by their ability to be recognized and targeted to peroxisomes by Pex19. Those that are targeted by Pex19 are called class 1 or mPTS1, and those that are not targeted by Pex19 are called class 2 or mPTS2 [129].

### A model for direct targeting to peroxisomes

**Fig. 2:**
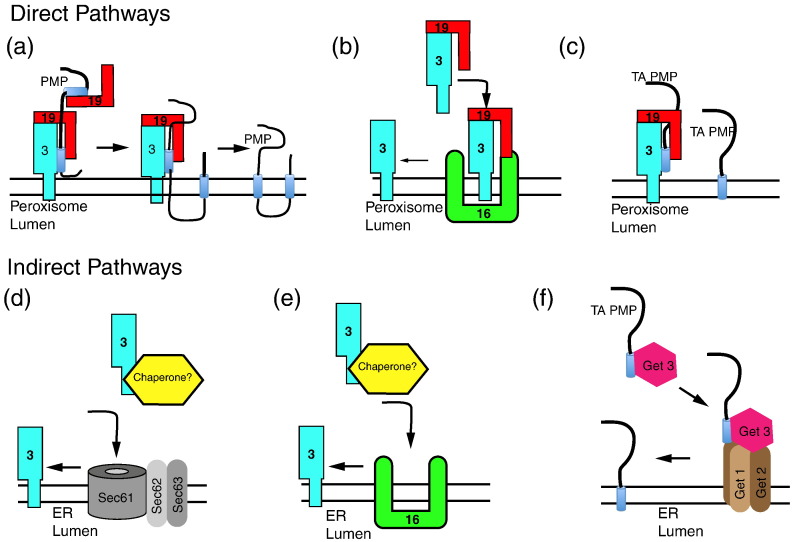
Models for PMP import pathways. PMPs are either inserted directly from the cytosol into the peroxisomal membrane (a–c) or inserted into the ER and subsequently sorted to peroxisomes (d–f). (a) The main mechanism of direct import of PMPs into peroxisomes dependent on Pex3 and Pex19. Pex19 acts as the cytosolic chaperone while Pex3 is the membrane anchor. Some PMPs have multiple mPTS sequences that likely bind multiple Pex19 to allow for the import of multi-membrane spanning PMPs. This mechanism seems universal. (b) Direct insertion of Pex3 into the peroxisomal membrane dependent upon Pex16 and Pex19 in mammals. (c) Similar to other PMPs, TA PMPs are inserted directly via the Pex3/Pex19 complex in mammals. (d) Insertion of Pex3 and other PMPs into the ER via the ER translocon. This process has been shown in *S*. *cerevisiae*. It is not known whether a cytosolic chaperone is required for this process. (e) In mammalian cells, Pex3 insertion into the ER requires Pex16. (f) TA PMPs in *S*. *cerevisiae* have been shown to use the Get complex. However, the mammalian homologue TRC40 complex is not involved in the import of TA PMPs.

The current model for direct posttranslational targeting of PMPs to peroxisomes proposes that Pex19 acts as a soluble recycling receptor/chaperone that picks up newly synthesized PMPs in the cytosol and subsequently docks on Pex3 in the peroxisomal membrane (Fig. 2a–c) [113], [138], [139]. In line with this model, Pex19 binds to newly synthesized PMPs to keep them soluble and stable in the cytosol. This is based on *in vitro* studies where Pex19 was found to bind newly synthesized PMPs and keeps them soluble [125], [126], [140], [141], [142]. In line with this, peroxisome-less cells (Pex3-deficient fibroblasts) overexpressing Pex19 stabilize PMPs in the cytosol and prevent them from being degraded [113], [143]. Furthermore, Pex19 not only binds PMPs but also transports them to different subcellular locations. For instance, the overexpression of Pex19 tagged with a nuclear localization signal resulted in the accumulation of PMPs in the nucleus [143]. Pex19 cannot insert, on its own, PMPs into the membrane; for this, the PMP membrane receptor protein Pex3 is also required. Together with the cargo, Pex19 binds to Pex3 on the peroxisomal membrane to form a trimeric complex of Pex3, Pex19 and cargo [125]. The importance of the Pex3–Pex19 interaction for PMP targeting is illustrated by mutations that prevent Pex3–Pex19 interaction that also prevents PMP insertion into peroxisomes [126], [144], [145].

Pex3 is anchored in the peroxisomal membrane by its N-terminal transmembrane domain, with the remainder of the protein forming an α-helical bundle that protrudes into the cytosol [144], [146]. This domain contains a hydrophobic surface located near the base of the protein close to the lipid bilayer. It is thought to mediate binding to liposomes *in vitro* [147] and has been suggested to locally deform the peroxisomal membrane [142]. Mutagenesis studies of this hydrophobic patch revealed the importance of its hydrophobicity and its shape for PMP insertion [145] but not Pex19 binding [142]. At the apex of the bundle lies a hydrophobic groove that mediates binding of an N-terminal amphipathic helix of Pex19 [144], [146]. The region on Pex19 that binds mPTSs is located in its C-terminal domain [138], [139], [148]. An additional amphipathic helix in Pex19 is thought to facilitate the handover of transmembrane domains to Pex3. Subsequent transmembrane insertion occurs where the Pex3 hydrophobic surface deforms the peroxisomal membrane [142].

PMPs with multiple transmembrane segments have multiple Pex19 binding domains: these PMPs may bind multiple Pex19s simultaneously and dock onto the same Pex3 sequentially or to multiple Pex3s (Fig. 2a) to allow their insertion into the bilayer.

## Indirect Targeting Pathway (via the ER)

The ER-to-peroxisome pathway was first described in *Yarrowia lipolytica* [149], [150]. One of the key observations was that Pex2 and Pex16 are core-glycosylated PMPs. This result suggested that these PMPs travel via the ER to peroxisomes bypassing the Golgi complex. No other endogenous PMPs have been shown to be glycosylated in any other model system, which is unfortunate as this would provide a useful tool to study ER to peroxisome transport. Since then, several PMPs have been described to travel to peroxisomes via the ER in a number of different systems, including ascorbate peroxidase and Pex16 in plants and Pex3 and Pex16 in mammals, as well as Pex3, Pex15 and Pex22 in *S*. *cerevisiae* [117], [151], [152], [153], [154], [155]. Of these PMPs, transport of Pex3 has been studied the most intensively.

Pex3, the protein that mediates direct insertion of PMPs into the peroxisomal membrane together with Pex19, is itself a PMP. This leads to an apparent conundrum of how Pex3 is itself inserted into the peroxisomal membrane. It has been known for some time that Pex3 targeting to peroxisomes differs from that of other PMPs as it contains an mPTS2 instead of an mPTS1 such as most PMPs [143]. This targeting quagmire was resolved by a number of groups who simultaneously described the transport of Pex3 via the ER to peroxisomes in *S*. *cerevisiae* [156], [157], [158]. In Pex3-deficient cells, conditional expression of Pex3-GFP allowed for the visualization of *de novo* peroxisome formation from the ER. Pex3 was seen first in the ER and associated puncta, after which the puncta lost their ER association and matured into peroxisomes [156]. Also, when Pex3 is forced to insert cotranslationally into the ER by addition of the invertase signal peptide, not only it complemented *pex3* cells but also, more importantly, the signal peptide was cleaved and the processed Pex3 was shown to end up in newly formed peroxisomes [157], thus showing that Pex3 traveled to peroxisomes via ER.

More recent studies suggest that Pex3 import into the ER in *S*. *cerevisiae* involves the ER posttranslational Sec61/Sec62/Sec63 translocon (Fig. 2d) [159]. The mPTS2 of Pex3 of *S*. *cerevisiae*, which was previously shown to be required for its peroxisomal localization [158], was dissected further into signals that direct Pex3 (1) into the ER, (2) sort it from there to a punctate ER subdomain (pER) and (3) sort it from the pER onto peroxisomes [159], [160].

Interestingly, Pex3 appears not to be the only PMP to be imported into the ER by the posttranslational translocon. For instance, partial depletion of the ER translocon subunits Sec62 and Sec63 in *S*. *cerevisiae* showed a partial mislocalization of Pex13-GFP to the cytosol [161]. Additionally, Pex13 and Pex14 fail to associate with membranes in *pex3* mutants depleted for Sec62/Sec63 as revealed by differential centrifugation [162]. Together, these observations make a strong case for a requirement of the Sec61 translocon for peroxisome biogenesis and membrane insertion of some PMPs in *S*. *cerevisiae*.

## Targeting of TA PMPs in *S*. *cerevisiae*: A Tale of Two Pathways

TA proteins destined for the endomembrane system insert into the ER membrane via a variety of partially redundant machineries [163], [164]. The ATP-dependent insertion via the Get pathway is the best studied of these mechanisms and appears to be a major contributor to insertion of TA proteins with a hydrophobic tail anchor region. The Get/TRC machinery consists of a cytosolic ATPase (Get3/TRC40) that binds the transmembrane segment of newly synthesized TA proteins and delivers them to the ER membrane [165], [166]. Get1 and Get2 are integral ER membrane proteins that function as Get3–cargo complex receptors in yeast [166].

Overexpression of the peroxisomal TA protein Pex15 results in its accumulation in the ER [166], [167]. In Get-deficient cells, a pulse of a GFP-Pex15 fusion protein fails to label peroxisomes and the ER but instead labels a cytosolic aggregate initially, which is followed by mitochondrial localization at later time points [166]. Pex15 appended with an opsin tag that is a 23-amino-acid residue is N-glycosylated and reaches peroxisomes [154]. Together, these observations indicate that Pex15 is initially targeted to the ER by the Get complex before being routed to peroxisomes. However, Pex15 can also reach peroxisomes independent of the Get machinery. In contrast to *pex15* cells [167], *get1*/*get2* and *get3* cells have peroxisomes [162], [166]. A short pulse of YFP-Pex15 expression showed low levels of YFP-Pex15 associated with peroxisomes in *get3* cells [162]. As Get3 and Pex19 have been shown to interact with the tail anchor region of Pex15 [166], [168], it is tempting to speculate that the direct pathway may also contribute to targeting *S*. *cerevisiae* Pex15 to peroxisomes.

In mammals and in *Neurospora crassa*, the Pex15 homologue, the TA PMP Pex26, is targeted and inserted independently of the Get/TRC machinery. Instead, it relies on the Pex19-dependent direct targeting pathway [126], [142]. Similarly, the TA protein Fis1 requires Pex19 and its Pex19 binding sites for targeting to peroxisomes [169].

These studies of TA proteins clearly show that this type of PMP can be targeted to peroxisomes using different machineries and pathways. In yeast, the predominant but not exclusive route appears to be the indirect pathway via the ER, whereas in mammalian cells and in *N*. *crassa*, the direct import into peroxisomes appears to dominate.

## Pex3 Targeting in Mammals

As observed in *S*. *cerevisiae* cells, human Pex3 fused to a signal peptide can complement Zellweger syndrome fibroblasts deficient in Pex3. This implies that, similar to yeast Pex3, human Pex3 can traffic to peroxisomes via the ER [153], [155]. However, using an *in vitro* targeting assay in semi-permeabilized cells, Fujiki's group has demonstrated that Pex3 can insert directly into peroxisomes by a pathway similar to that shown for other PMPs in that it requires the PMP cytosolic receptor Pex19. Since Pex3 lacks an mPTS1 but instead contains a targeting signal that does not binds Pex19 [143], the role of Pex19 in this transport is unclear, but may reflect its chaperone function.

Pex3 targeting directly to peroxisomes also requires Pex16, which is considered to function as a peroxisomal receptor for newly synthesized Pex3 (Fig. 2e) (Matsuzaki, 2008). Pex16 has a dual localization as it is also found in the ER membrane [117]. In live cells, Pex3 was shown to target to the ER when Pex16 was localized there even in cells deficient in functional Pex19 (Fig. 2b) [117], [145]. However, in the absence of Pex16, Pex3, like many other PMPs, localized to mitochondria even in the presence of Pex19 [117], [143], [153]. Furthermore, in addition to Pex3, a number of other PMPs are recruited to the ER by Pex16 [117], [155]. These data suggest a model whereby Pex3 and maybe some additional PMPs are targeted directly to peroxisomes via a Pex16-dependent, Pex19-dependent route, as well as to the ER by a Pex16-dependent, Pex19-independent route. The steady-state distribution of Pex16 and the availability of Pex19 binding sites on cargo molecules may determine which route is taken in mammalian cells.

How Pex16 itself is imported into a membrane is a point of contention. It has been reported that Pex16 is imported by the Pex3/Pex19 direct pathway [143], while another group suggests that Pex16 is cotranslationally inserted into the ER membrane before being routed to peroxisomes [117]. The different conclusions may result from differences in the Pex16 constructs used. The report showing direct targeting of Pex16 to peroxisomes is based largely on the ability of Pex19 to bind and redirect the last third of the Pex16 polypeptide to the nucleus and peroxisomes. However, the first half of Pex16 has been shown to be necessary and sufficient to target to peroxisomes in control cells [133]. *In vitro* targeting experiments suggest that Pex16 is inserted cotranslationally into the ER membrane [117], most likely by the ER translocon. Neither Pex3 nor Pex19 is required for its ER insertion. Furthermore, recent work that quantified PMP import kinetics showed that the import rate of Pex16 into peroxisomes was low compared to that of Pex3 and PMP34. It is proposed that direct import into peroxisomes is faster than insertion into the ER membrane and subsequent sorting to peroxisomes. Indeed, Pex16 transport kinetics are similar to those of Pex3 fused to a signal peptide, a construct that is first forced into the ER by an ER targeting signal sequence before being routed to peroxisomes [155]. It is also possible that mammalian Pex16 has two independent targeting sequences, one in the N-terminal part of the protein for cotranslational insertion into the ER membrane and one in the C-terminal part of the protein for direct insertion into the peroxisomal membrane. Since the ER targeting signal will emerge first from the ribosome, we propose that most Pex16 is cotranslationally inserted into the ER.

## ER-to-peroxisome transport

Since it is clear that some PMPs travel via the ER to peroxisomes, this raises the question as to how this transport is mediated. Two models explain how ER-inserted PMPs reach peroxisomes. In the first model, ER-inserted PMPs are sorted away from the secretory pathway and are delivered to peroxisomes, probably by vesicular transport. In the second model, peroxisomes form *de novo* from ER-derived vesicles thereby forming a new peroxisome. The two processes may be combined into a single process where a pre-peroxisomal vesicle either matures into a new peroxisome or fuses with an existing peroxisomes. Which process dominates may vary among growth conditions, cell types or whether any peroxisomes are present.

In mammalian cells, the first model is supported by the finding that Pex16 is inserted into the ER membrane and sorted from there to peroxisomes [117], [170]. This transport requires the secretory factor Sec16b [170]. Similarly, Pex3 “forced” into the ER by a signal sequence is transported to all pre-existing peroxisomes in mammalian cells [155]. This suggests that the ER is constantly providing lipids and proteins to pre-existing peroxisomes. The rate of transport of ER-targeted Pex3 was quite high, thus giving a possible explanation as to why PMPs are not readily found in the ER in wild-type cells. Further support for a model whereby existing peroxisomes continue to receive membrane material was provided by overexpression of a Pex11β-YFP fusion protein that inhibits peroxisome fission. Newly synthesized matrix and membrane proteins are imported into subdomains of these elongated peroxisomal structures [171]. Additionally, using Halo-tag approaches, the Fransen laboratory showed that peroxisomes receive newly synthesized PMPs, including Pex16 (Huybrechts *et al.*, 2009).

In cells that are devoid of peroxisomes due to loss of PMP import peroxins (Pex3 or Pex19), peroxisomes can form *de novo* on introduction of the corresponding gene. In *S*. *cerevisiae*, Pex19 is required for exit of Pex3 from the ER [156], [158], [172]. *In vitro* reconstitution of the initial step of *de novo* formation revealed that Pex19 is required for release of Pex3-containing vesicles from the ER [154], [173]. However, how Pex19 mediates PMP targeting and budding from the ER is unclear. It is possible that Pex19 mediates recruitment to Pex3 at the ER of factors required for pre-peroxisomal vesicle formation. This may not be mechanistically different from the role of Pex19 in PMP targeting. For instance, in *S*. *cerevisiae*, Pex25, a member of the Pex11 family, has been reported to work with Pex3 to initiate formation of pre-peroxisomal vesicles [174]. As Pex25 is an integral membrane protein, Pex19 may play a role in delivering it to Pex3 at the ER. Pex19 may also be involved in delivering to the ER factors required for vesicle formation. In general, vesicle budding from donor membranes involves recruitment of peripheral membrane proteins from the cytosol. Instead of handing the cargo over to Pex3 for insertion, peripheral membrane proteins may be handed over to other factors at the pre-peroxisomal membrane. Indeed, Pex19 has been shown to recruit the peripheral membrane proteins Vps1, Pex17 and Myo2 to peroxisomes via a motif resembling the mPTS1 [175], [176], [177].

Recently Tabak and colleagues proposed that, in *S*. *cerevisiae*, all newly synthesized PMPs insert into the ER membrane via the ER translocon or Get complex. The PMPs then sort to distinct ER subdomains that bud off and heterotypically fuse to bring together a complete set of PMPs, after which matrix protein import commences [161], [162]. The peroxins Pex1 and Pex6 are proposed to mediate vesicle fusion. The import defect observed in *pex1* and *pex6* cells is proposed to result from a lack of fusion. According to this model, existing peroxisomes do not receive newly synthesized PMPs and therefore there is no need for a PMP insertion machinery in the peroxisomal membrane.

Although this model is attractive, it does not explain the strong experimental evidence that existing peroxisomes continue to receive newly synthesized PMPs and that peroxisomes multiply by dynamin-related protein fission [160], [172], [178], [179], [180]. Furthermore, since many *S*. *cerevisiae* PMPs contain Pex19 binding sites that are required for their targeting [137] and since the steady-state localization of Pex3 is at the peroxisomal membrane, it is very likely that, by analogy to mammalian cells, most PMPs are inserted directly into peroxisomal membranes. Pex3 that travels via the ER to peroxisomes may facilitate PMP insertion into the ER and into subsequent transport intermediates along the Pex3 trafficking pathway. The kinetics of ER to peroxisome transport and the availability of Pex3 for PMP import will determine whether newly synthesized PMPs are imported directly into peroxisomes or indirectly via the ER. This hypothesis is in line with a recent observation by the van der Klei group. In *H*. *polymorpha* cells lacking Pex3, non-ER membrane structures containing the PMPs Pex13 and Pex14 but lacking other PMPs were detected [118]. Upon reintroduction of Pex3, Pex3 accumulated in these structures and subsequently import of other PMPs commenced. Therefore, in this yeast, it is the localization of Pex3 that appears to direct the site of import.

The proposal that Pex1 and Pex6 are involved in fusion of precursor peroxisomes [161] is difficult to reconcile with previous studies. According to this model, the matrix protein import defect observed in *pex1* and *pex6* cells is a consequence of the docking complex (Pex13/Pex14) being present in separate vesicles from the exportomer complex (including Pex2/Pex10/Pex12). Many independent studies in various model systems have shown that Pex1 and Pex6 are required for recycling of ubiquitinated PTS receptors (see above). In *pex1* and *pex6* cells, PTS receptors accumulate at a postdocking stage at the peroxisomal membrane and are poly-ubiquitinated by the transmembrane RING finger ubiquitin ligases that are part of the exportomer complex, before their degradation. These observations indicate that docking complex and exportomer are present in the same membrane structures in *pex1* and *pex6* cells. On the other hand, in support of Tabak's model, *Y*. *lipolytica* Pex1 and Pex6 have been suggested to function in membrane fusion events. Clearly, these observations are not easily explained and further investigations will be needed to resolve this apparent contradiction.

## Concluding Remarks and Considerations

Although our understanding of peroxisome biology has been much advanced, there remain several huge gaps in our knowledge and in some unexplained observations. One such gap is the mechanism by which matrix proteins and membrane proteins are translocated across/into the peroxisomal membrane. A translocon for matrix proteins has been identified, but its transient and dynamic nature has hampered detailed biochemical characterization. Innovative approaches will be required to unravel the mysteries of translocation into the peroxisomal matrix.

With respect to delivery of PMPs to peroxisomes, there is evidence for two routes, one direct and one indirect via the ER. A particular PMP may follow different routes to peroxisomes but the factors that decide which route it will follow are not clear. Mechanistic insights are limiting and it is entirely unclear how PMPs with multiple membrane spans are inserted into the peroxisomal membrane.

Other major gaps in our understanding include the mechanisms that regulate peroxisome dynamics. What determines whether peroxisomes multiply via growth and division or via *de novo* formation from the ER, and what are the signals for peroxisome degradation? Are there new machineries to be discovered or are all the peroxins known? Genetic approaches initially identified peroxins as factors for matrix protein import or membrane biogenesis. However, it is becoming clear that many peroxins have multiple functions. For instance, Pex3 has been implicated in peroxisome segregation and pexophagy in various yeast species [181], [182], [183]; Pex19 functions in peroxisome fission and segregation [175], [177], and Pex14 has been implicated in pexophagy and peroxisome motility [184], [185], [186]. As discussed above, the exportomer factors Pex1 and Pex6 have been proposed to also mediate fusion events during peroxisome formation in *Y*. *lipolytica* and *S*. *cerevisiae* [161], [187].

With many peroxins being involved in a variety of aspects of peroxisome dynamics and with different modes of peroxisome multiplication and various routes for PMP targeting that may display partial overlap, it is not surprising that studies on peroxisomal membrane biogenesis have resulted in controversies and unexplained observations. Careful dissection of the different functions of peroxins under controlled growth conditions is an important avenue to follow and may unravel the mechanisms that coordinate various aspects of peroxisome dynamics and resolve some of the unexplained observations discussed above.

For the last 20 years, the research focused on understanding peroxisome biogenesis has advanced the field significantly. However, several studies clearly demonstrate the need to understand the degradation process, as it appears that the cell has an intricate process of detecting and destroying misassembled peroxisomes [118], [188]. This indicates that caution must be taken when interpreting data using peroxisome biogenesis mutants, as increased turnover of peroxisomes and/or pre-peroxisomal structures can potentially skew the results. Only by understanding both processes may we be able to comprehend better the mechanisms governing peroxisome dynamics.

## Figures and Tables

**Table 1:** Peroxins required for peroxisomal matrix protein import in *S*. *cerevisiae* and their human orthologues

*S*. *cerevisiae*	*Homo sapiens*	Process	Complex
Pex1	Pex1	Export of receptors	AAA + complex
Pex2	Pex2	Ubiquitination of receptors	RING ubiquitin ligase complex
Pex4	UbcH5a/UbcH5b/UbcH5c	Receptor ubiquitination	Ubiquitin conjugation complex
Pex5	Pex5S/Pex5L	Targeting, translocation	PTS1 receptor, import pore
Pex6	Pex6	Export of receptors	AAA + complex
Pex7	Pex7	Targeting	PTS2 receptor
Pex8		Translocation/cargo release	Intraperoxisomal peroxin
Pex10	Pex10	Receptor ubiquitination	RING ubiquitin ligase complex
Pex12	Pex12	Receptor ubiquitination	RING ubiquitin ligase complex
Pex13	Pex13	Docking	Docking complex
Pex14	Pex14	Docking, translocation	Docking complex, import pore
Pex15	Pex26	Export of receptors	AAA + complex
Pex17		Docking	Docking complex
Pex18/Pex21	Pex5L	Targeting	PTS2 coreceptor
Pex22		Receptor ubiquitination	Ubiquitin conjugation complex
